# Supracrestal Complex Prosthetic Modification and Topical Oxygen Therapy for Peri-implant Mucositis Resolution in an Infrapositioned Implant: A Case Report With Three-Year Follow-Up

**DOI:** 10.7759/cureus.90181

**Published:** 2025-08-15

**Authors:** Minas Leventis, Ioanna Mitsika, Ioannis Vergoullis, Konstantinos Valavanis

**Affiliations:** 1 Oral Surgery, Dental School, National and Kapodistrian University of Athens, Athens, GRC; 2 Dentistry, National and Kapodistrian University of Athens, Athens, GRC; 3 Periodontics, Louisiana State University School of Dentistry, New Orleans, USA; 4 Oral Surgery, Dental School, University of Naples Federico II, Naples, ITA

**Keywords:** abutment modification, craniofacial growth, dental implants, implant complications, implant supracrestal complex, infraposition, oxygen therapy, peri-implant mucositis, soft tissue seal

## Abstract

Long-term implant success depends on stable integration with peri-implant tissues and adequate bacteria control. However, implants placed in adolescents may later exhibit prosthesis infrapositioning and soft tissue complications due to continuous craniofacial growth. A 30-year-old healthy woman presented with long-standing inflammation around an implant-supported crown at site #23, placed at age 18. Clinical evaluation revealed deep probing depth (7 mm), bleeding on probing, and signs of cement-induced inflammation. The crown was infrapositioned and exhibited poor marginal adaptation, subgingival porcelain exposure, and residual cement. Despite these complications, crestal bone levels were preserved. The prosthesis was removed and replaced by a chairside-fabricated provisional crown composed of a highly polished composite. The subgingival portion of the provisional crown was thoroughly designed to allow for peri-implant soft tissue re-adherence and preservation and support of the peri-implant soft tissue topography, while the topical use of oxygen- and lactoferrin-releasing oral gel aimed at enhancing the soft tissue healing, reducing inflammation, and controlling the pathogenic bacteria. After healing, a final screw-retained implant crown was delivered, fulfilling biological, functional and aesthetic requirements. At the three-year follow-up, the peri-implant tissues were clinically healthy, with probing depths less than 3 mm and no bleeding. Inflammation was resolved, soft tissue architecture remained stable, and radiographic bone levels were unchanged. This case report illustrates the successful management of peri-implant complications linked to prosthesis infrapositioning and poor prosthetic design, following a biologically oriented approach that combined supracrestal complex management and topical oxygen therapy.

## Introduction

The symbiotic interaction between the implant, the peri-implant tissues and the oral biofilms constitutes a complex biological system characterized by several fundamental pillars: soft and hard tissue quality and quantity, implant design and three-dimensional position, abutment and prosthesis design, host response and bacteria control. These important elements do not function in isolation; they are highly interconnected and interdependent, forming a dynamic framework responsible for favorable to peri-implant health, thus determining the level of success and long-term stability of the implant [1].

Although dental implants constitute the preferred option for the replacement of missing single teeth, considering aesthetic, biological and functional parameters and due to their documented high survival rate, in children and adolescents, they should be planned with caution or postponed till growth is completed [2]. Considering that implants act as ankylosed teeth, they don't follow the growth pattern of the surrounding hard and soft tissues, failing to adapt to positional changes of the natural adjacent teeth. Thus, placing implants in growing jaws is possible to result in infrapositioning of the fixtures in the arch, leading to serious complications. Treatment strategies should be personalized according to the characteristics of each case, including simple retention, adjustment or replacement of the implant restoration, surgical implant repositioning by segmental osteotomy combined with osseodistraction, submergence or removal of the implant [3].

This paper aims to present a case where the chief concern of the patient was the persistent peri-implant mucositis around the prosthesis of an infraoccluded maxillary canine implant. The excessively deep implant position, the inadequate prosthesis design and the lack of efficient plaque control played a decisive role in the establishment of long-standing clinical complications. A comprehensive approach for the proper management and resolution of the problem is presented and analyzed, focusing on and highlighting the crucial role of implant positioning, design and material selection of the implant restoration, and biofilm control.

## Case presentation

A 30-year-old female patient presented with persistent inflammation around the implant-supported prosthesis at site #23. The patient was medically healthy with no history of systemic disease, medication use, allergies, tobacco, or vaping. An evaluation of the patient's dental history revealed that site #23 had been rehabilitated with a dental implant (Astra Tech, Dentsply Implants Manufacturing GmbH, Hanau, Germany) at the age of 18 years.

A comprehensive periodontal examination revealed localized gingivitis, and the gingival biotype was classified as thin. Plaque and bleeding indices were both below 10%, indicating an acceptable oral hygiene level.

**Figure 1:**
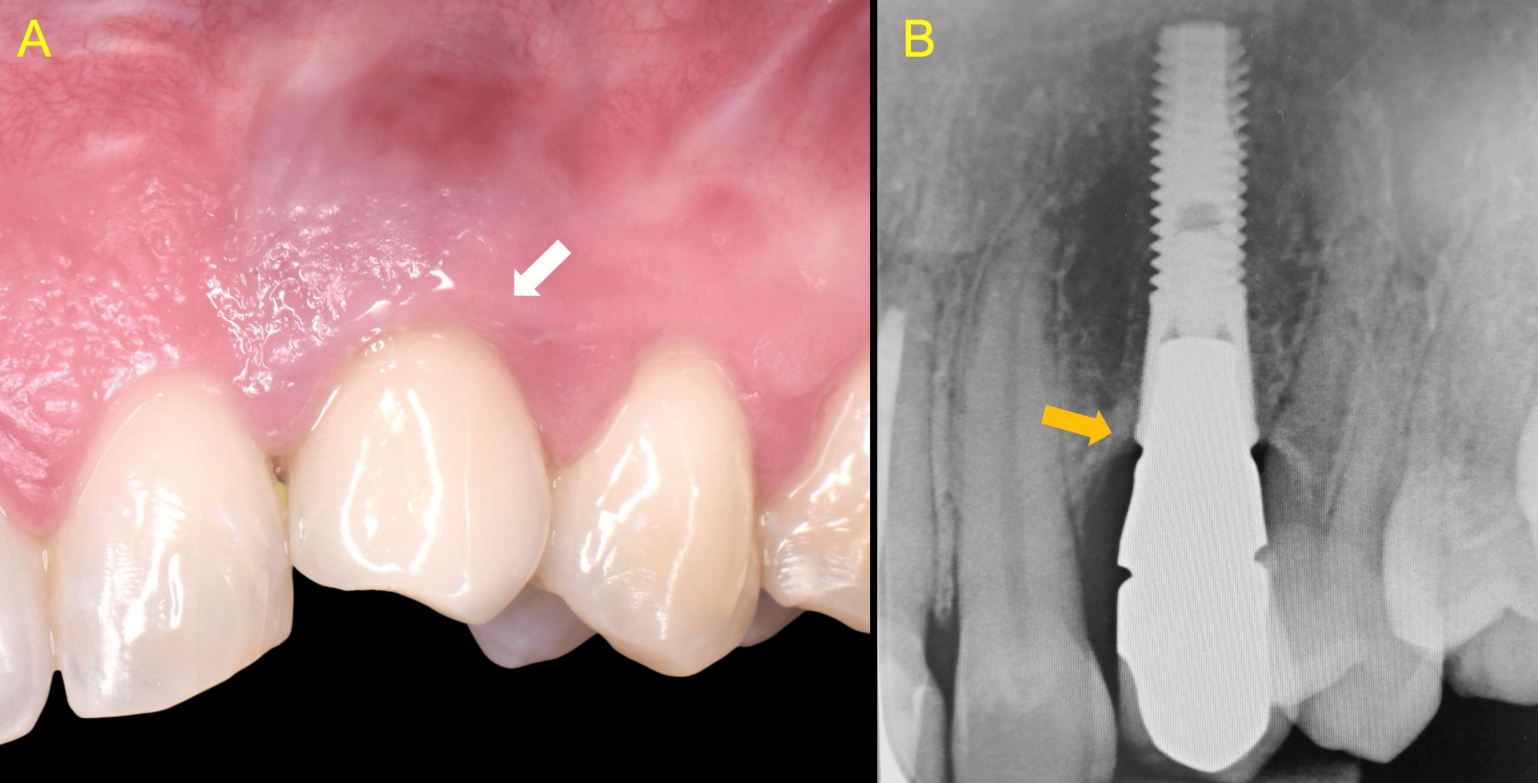
Continued skeletal growth resulted in partial submergence of the implant crown into the soft tissues A: Clinical view showing partial submergence of the implant crown into the soft tissues (white arrow) B: Periapical radiograph revealing an excessively deep implant platform with insignificant crestal bone loss (orange arrow)

Clinical and radiographic examination of site #23 showed that the implant platform was located excessively deep and the prosthesis was infrapositioned. These findings were attributed to continued skeletal growth, which had led to partial submergence of the implant crown into the soft tissues. A periapical radiograph revealed stable bone margins around the implant, with insignificant crestal bone loss (Figure 1).

**Figure 2:**
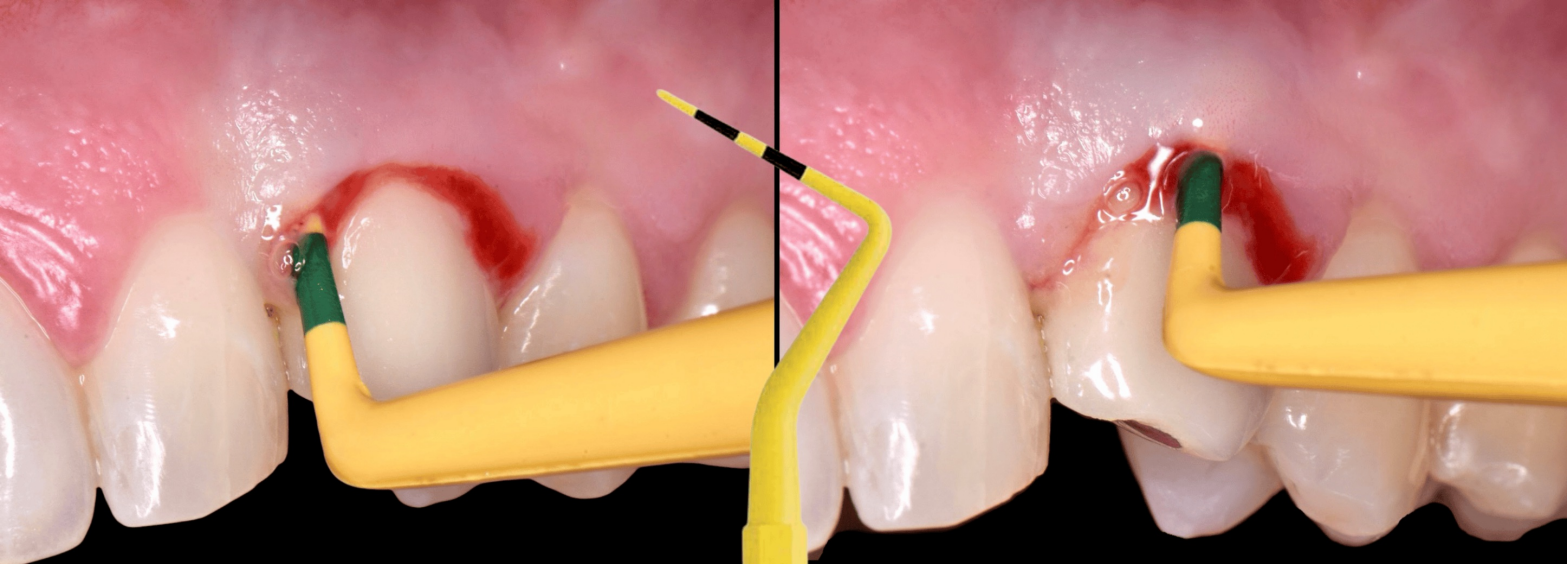
Bleeding on probing and 6-7 mm pocketing with hemorrhagic and purulent discharge

The migration of the zenith of the crown subgingivally resulted in chronic soft tissue inflammation due to the inability to properly control plaque and the presence of non-biocompatible porcelain beneath the gingival margin. Probing of the peri-implant sulcus revealed circumferential pocket depths of 6-7 mm, with severe bleeding, accompanied by hemorrhagic and purulent discharge (Figure 2).

**Figure 3:**
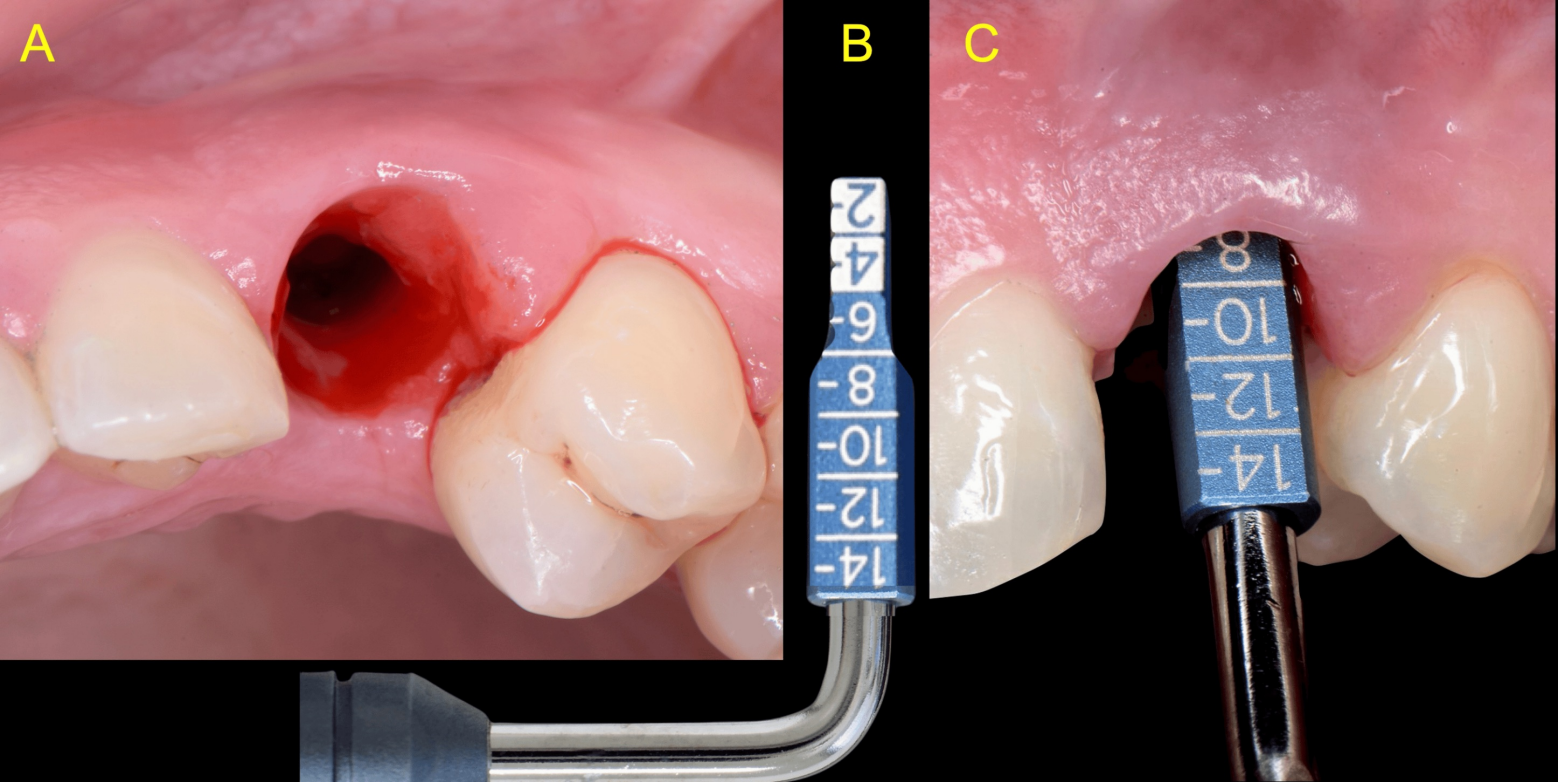
Measurement of the distance from the implant platform to the soft tissue zenith A: Clinical view of the supracrestal soft tissue topography B: Cervico caliper used for measurement C: The vertical distance was recorded as 7mm

The implant crown was removed to allow thorough inspection of the supra-structure and peri-implant tissues. Using a caliper (Cervico Caliper, VP Innovato Holdings Ltd, Cyprus), the vertical distance from the implant platform to the soft tissue zenith was measured at 7 mm mid-buccally, corresponding to the previously recorded probing depth (Figure 3).

**Figure 4:**
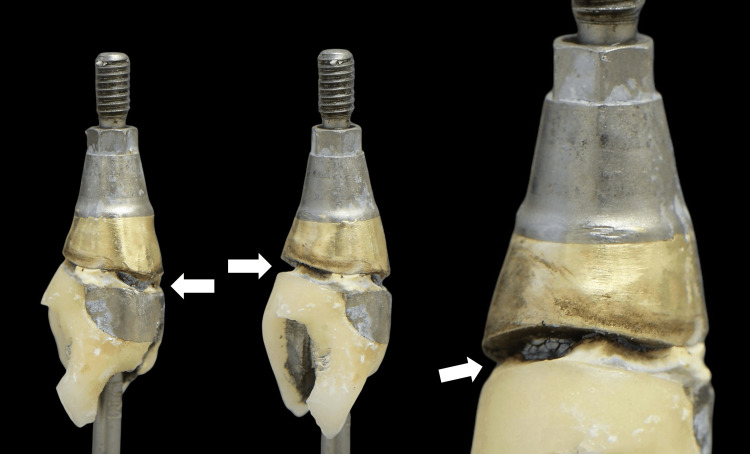
Removal of the implant restoration After drilling through the porcelain and metal framework, the prosthetic screw access channel was located, and the restoration was removed. Open margins and remnants of cement were observed deep subgingivally (arrows)

This finding dictated the absence of any mucosal barrier present above the implant platform. Upon inspection, the retrieved restoration was found to have a poor marginal fit with residual excess cement (Figure 4).

Based on the clinical and radiological findings, a diagnosis of peri-implant mucositis and cement sepsis (cement-induced inflammation) was established. The treatment plan involved thorough disinfection of the site, promotion of the healing of the soft tissues, and prosthesis replacement, which the patient consented to.

**Figure 5:**
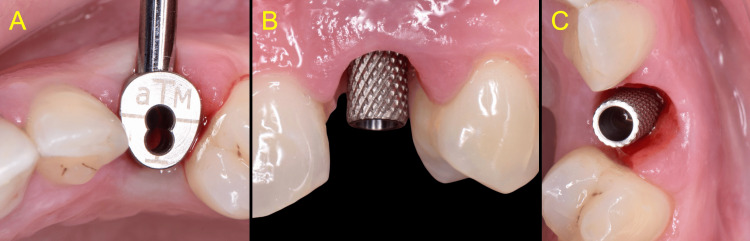
Initial clinical steps in the fabrication of the customized anatomical healer A: Intraoral assessment of the cervical profile using the appropriate anatomical tab from the Cervico Guide. In this case, the medium-sized (#M) anatomical tab of the anterior group (#a) matched the soft tissue topography at the cervical level B, C: A stock titanium temporary abutment was utilized for the subsequent chair-side customization to create the anatomical healer

The area was thoroughly irrigated with sterile saline, and the internal surface of the soft tissues was de-epithelialized using a diamond round bur under copious irrigation. The site was then treated for five minutes with an oxygen- and lactoferrin-releasing oral gel (blue®m, Wapenveld, Netherlands), followed by sterile saline rinsing. A cervical tissue profiler guide (Cervico Guide, VP Innovato Holdings, Cyprus) was used to determine the appropriate shape and dimensions for a customized anatomical healer (Figure 5).

**Figure 6:**
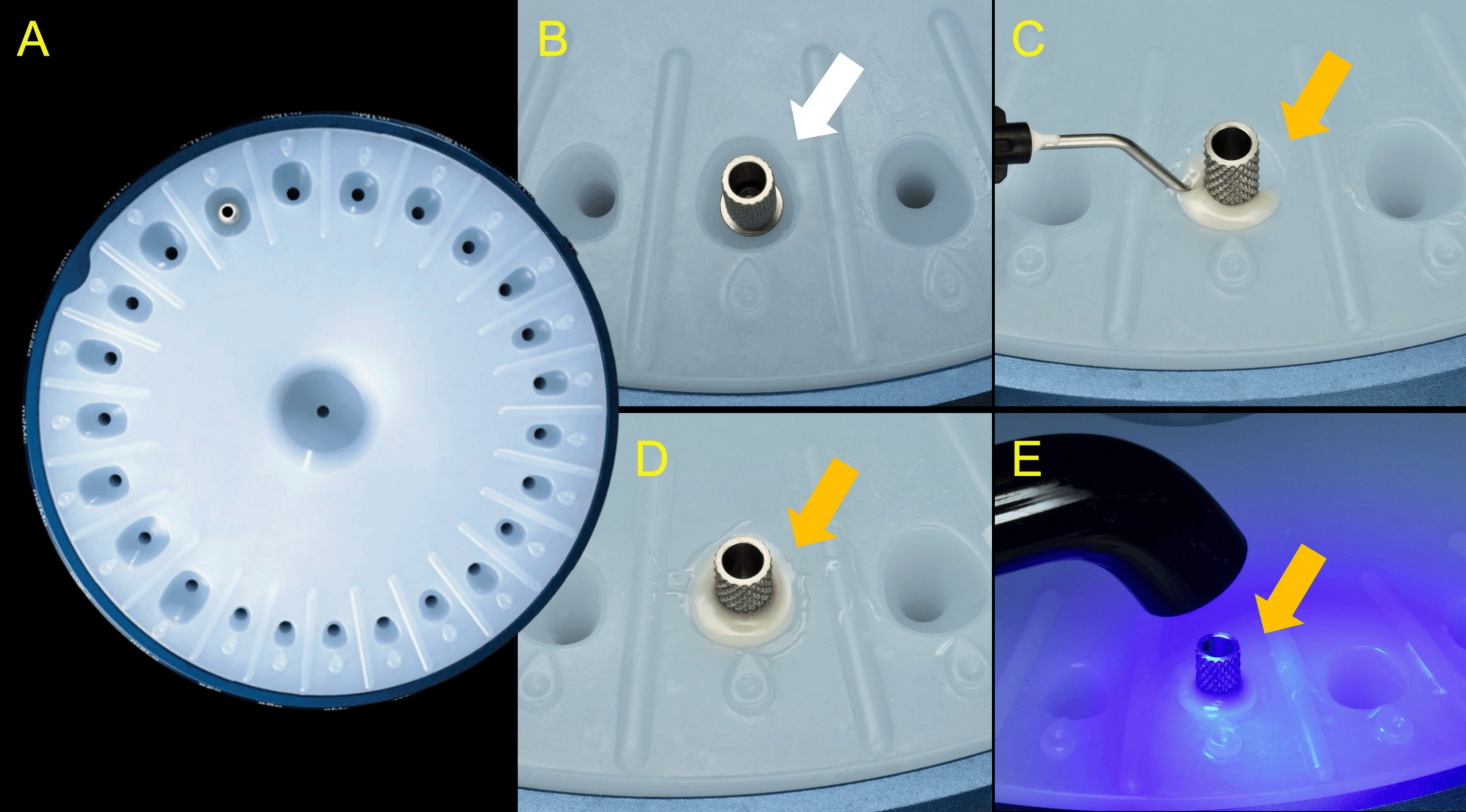
Chair-side fabrication of the customized anatomical healer A: The Cervico Essential Mold was utilized for abutment customization B. The stock titanium cylinder was fitted into the corresponding silicon well (white arrow) C-E: Nano-hybrid flowable composite resin was gradually injected around the titanium cylinder, and light-cured to create the anatomical sub-gingival portion of the abutment (orange arrows)

This was fabricated chairside using a dedicated mold (Cervico Essential Mold, VP Innovato Holdings, Cyprus), a stock titanium cylinder (Astra Tech, Dentsply Implants Manufacturing GmbH, Hanau, Germany), and a nano-hybrid flowable composite resin (Purefill Bio+, ELSODENT, France) (Figure 6). 

**Figure 7:**
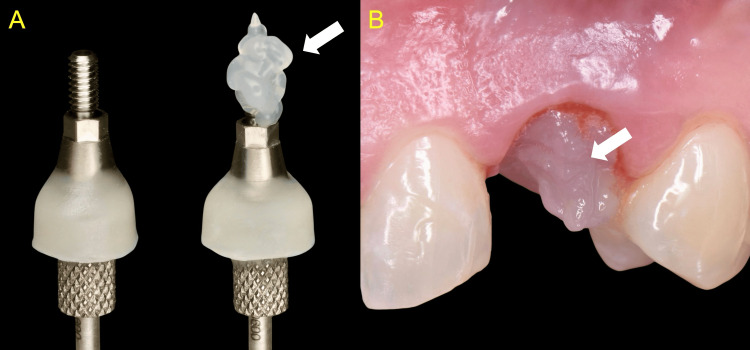
Application of oxygen- and lactoferrin-releasing gel (A) The customized anatomical healer (B) Both the customized anatomical healer and the site were treated with blue®m oral gel prior to installation (arrows)

The generated anatomical healer serves as a supracrestal structure designed to fulfil the biological and anatomical principles of soft tissue integration. The deep zone is formed by the concave titanium shoulder, while the transitional and cervical zones were provided by the highly polished composite resin. A small amount of blue®m gel was applied in the area and onto the anatomical healer, which was then installed onto the implant (Figure 7).

**Figure 8:**
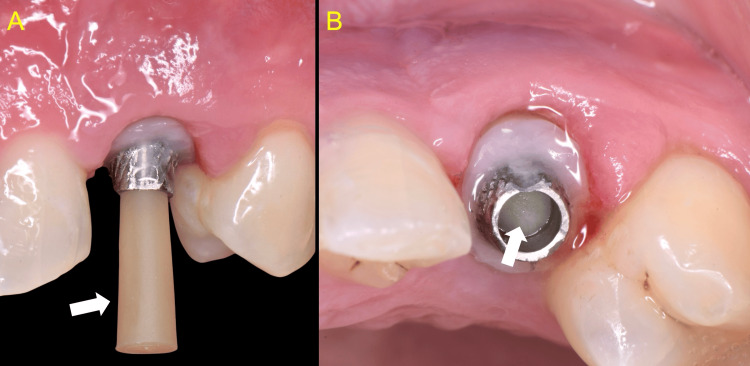
An antibacterial SilverPlug (A) was appropriately cut and introduced in the prosthetic canal (B) (arrows) SilverPlug: SilverAid, Italy

The gel delivers a safe, controlled release of active oxygen and lactoferrin, offering anti-microbial, anti-inflammatory, and healing-promoting effects. The anatomical abutment was torqued in accordance with the manufacturer's recommendations. The prosthetic screw access channel was sealed with a silver-containing antibacterial polymer cone (SilverPlug, SilverAid, Italy), trimmed to size, and overlaid with a top layer of flowable composite resin (Figure 8).

**Figure 9:**
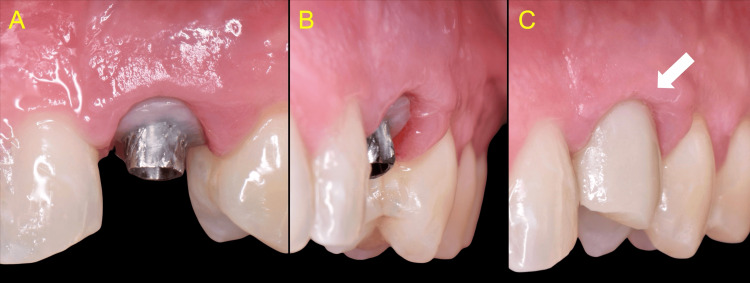
Clinical outcome following the placement of the customized anatomical abutment A, B: The margin of the chair-side fabricated abutment is now located above the soft tissue zenith, thereby creating favorable conditions for resolution of the inflammation and optimal soft tissue healing C: A provisional acrylic crown was placed using temporary cement (arrow)

An acrylic crown with supra-gingival margins was fabricated and cemented with temporary zinc oxide dental cement (TEMP BOND (NE), Kerr) onto the healer (Figure 9).

Detailed instructions on oral hygiene were provided to the patient, including rinsing with blue®m oxygen- and lactoferrin-releasing mouthwash, followed by blue®m oral gel application to the peri-implant area twice daily for post-operative local infection control and promotion of soft-tissue healing.

**Figure 10:**
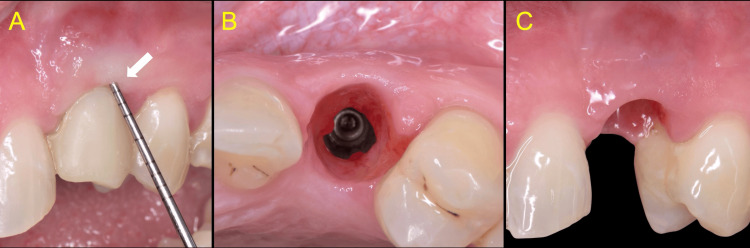
One-month review A: Absence of soft tissue inflammation. The epithelium is already attached subgingivally on the highly polished composite. One mm probing depth (arrow) B, C: Healthy peri-implant soft tissues, and stable topography

The patient was re-evaluated at 2-, 4-, and 12-week post-treatment, with the healing process progressing uneventfully. At the one-month clinical examination, periodontal probing revealed no signs of inflammation or pocketing. These findings suggest the possible establishment of epithelium attachment onto the highly polished composite surface of the healer. Upon removal of the temporary prosthesis, a more detailed assessment confirmed the absence of inflammation and the maintenance of the peri-implant soft tissue topography (Figure 10).

**Figure 11:**
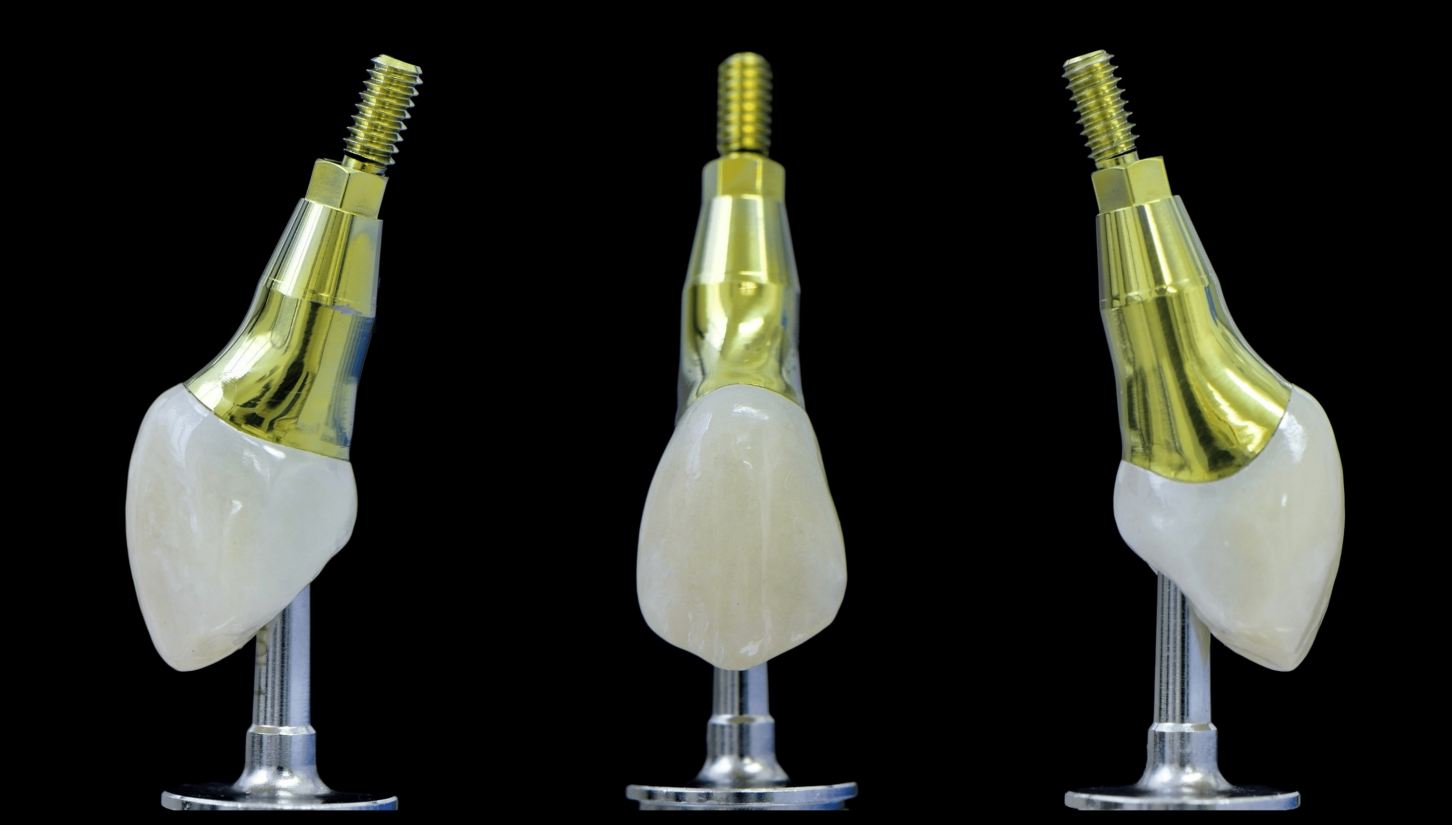
Final prosthesis Screw-cemented design, monolithic, zirconia crown permanently cemented on a milled titanium abutment

At this point, digital recordings were obtained, and the generated standard triangular language (STL) files were forwarded to the dental laboratory for the design and fabrication of a new final prosthesis (Figure 11).

**Figure 12:**
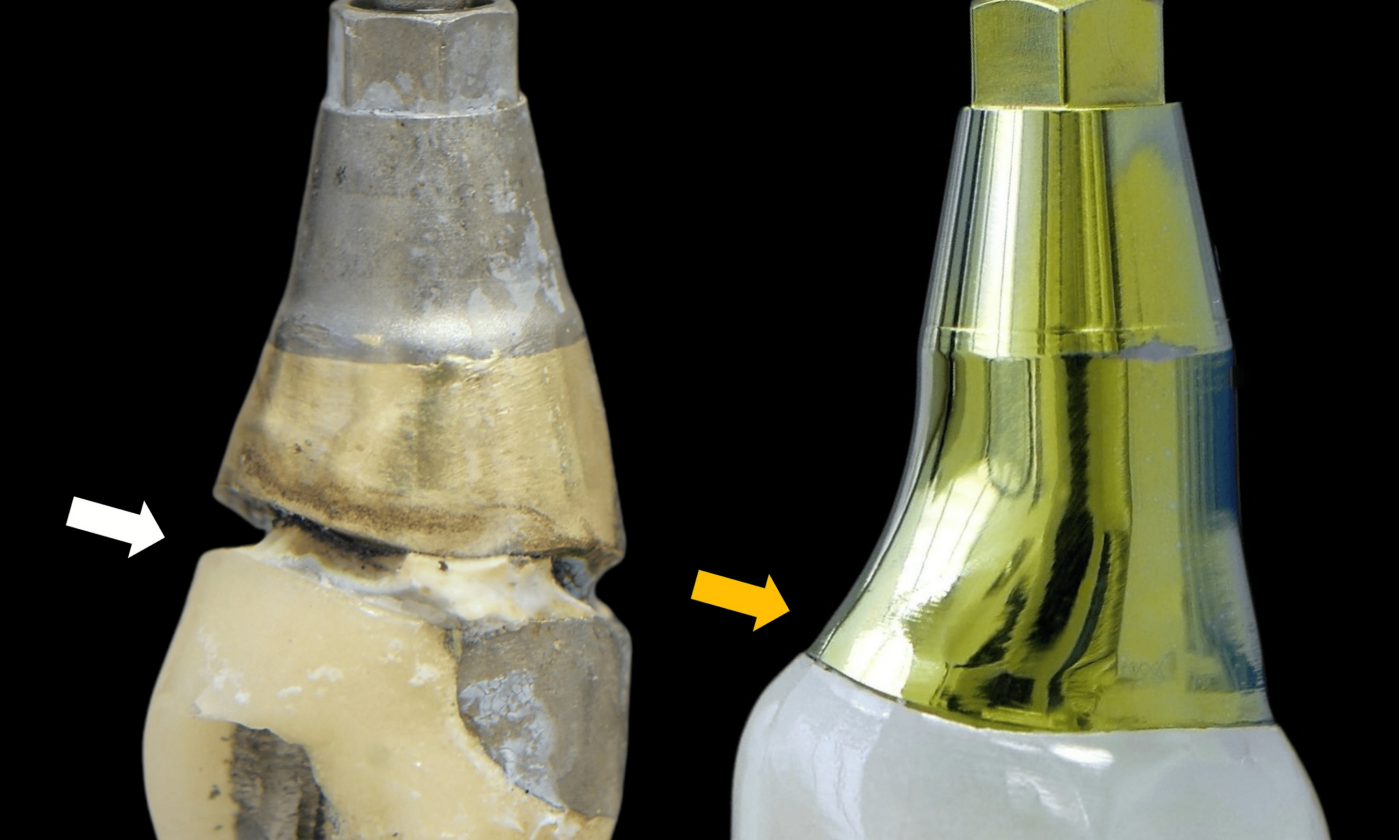
Comparison between the old (white arrow) and the new (orange arrow) implant restorations The use of CAD/CAM workflow enabled the fabrication of an anatomical abutment and crown with a natural emergence profile and a proper spatial outline at the cervical margin, precisely matching the existing soft tissue anatomy. The new restoration (orange arrow) incorporates biocompatible materials that support soft tissue attachment on, contributing to the essential formation of a stable peri-implant soft tissue barrier

Following the principles of biologically designed zones, a custom titanium abutment comprising a 4mm height shoulder was milled, and a monolithic zirconia crown was fabricated and subsequently cemented extra-orally onto the abutment. Particular attention was given to the subgingival portion of the crown to be highly polished and not glazed (Figure 12).

**Figure 13:**
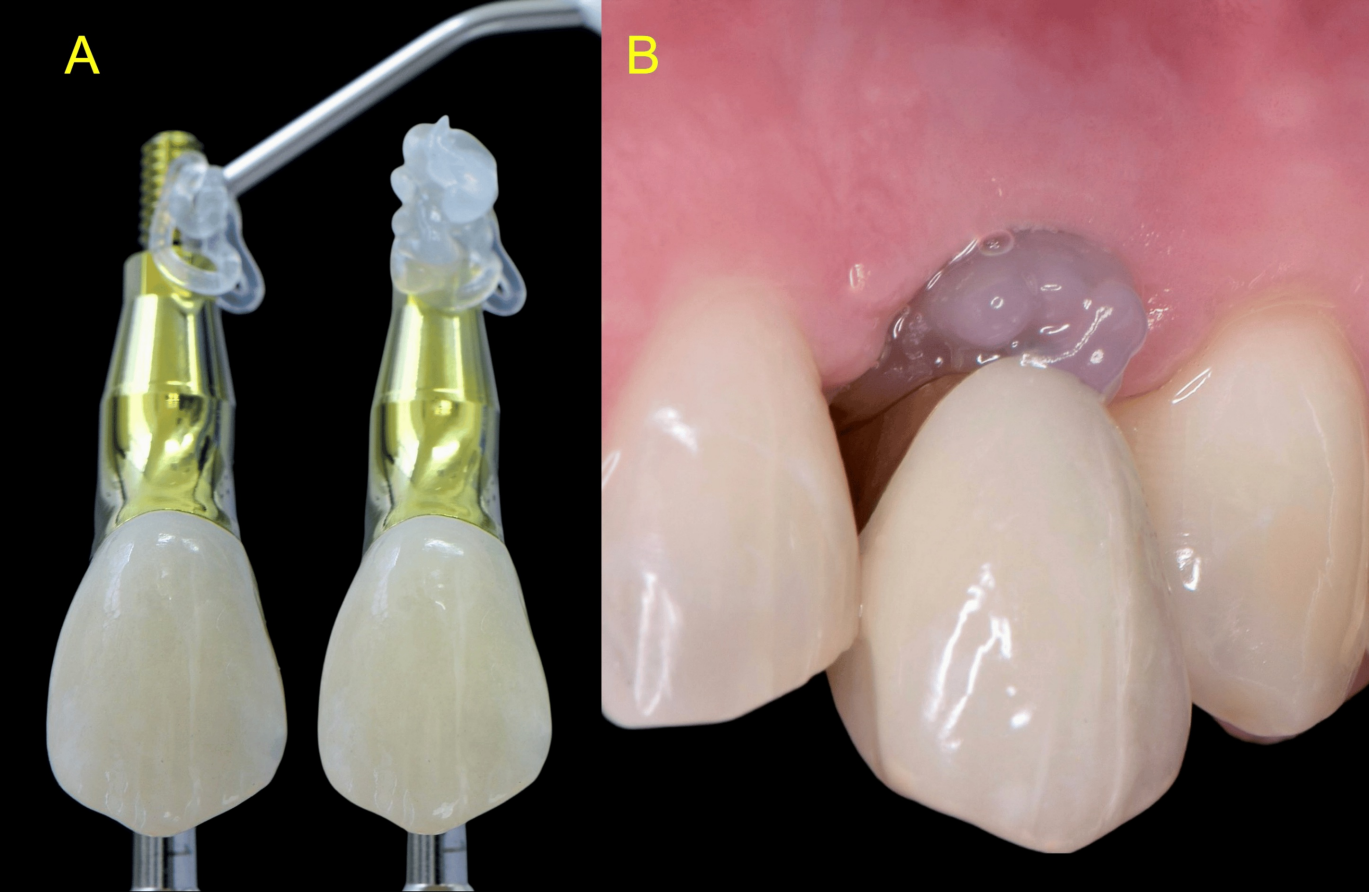
Application of oxygen- and lactoferrin-releasing blue®m gel (A) at the fitting of the final restoration (B)

Upon prosthesis delivery, blue®m was applied to the abutment of the final restoration (Figure 13). 

**Figure 14:**
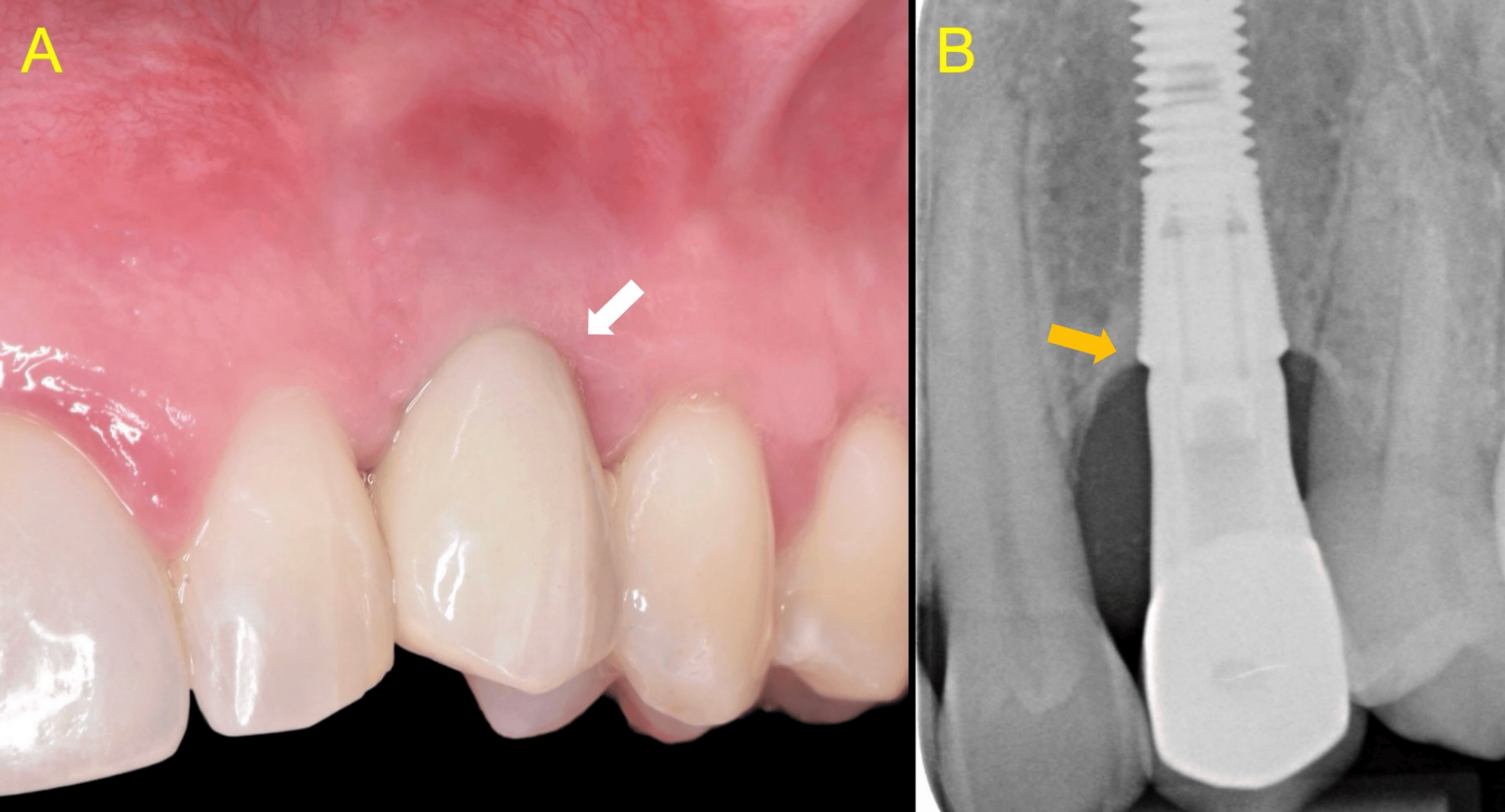
Evaluation at fitting of the final restoration A: Clinical view demonstrating a favourable aesthetic outcome with healthy peri-implant soft tissues (white arrow) B: Periapical radiograph showing the accurate fit of the implant crown (orange arrow)

Clinical and radiographical evaluation confirmed the accurate fit of the supra-structure, and the prosthesis was torqued to 35 N/cm, in accordance with implant manufacturer specifications (Figure 14).

**Figure 15:**
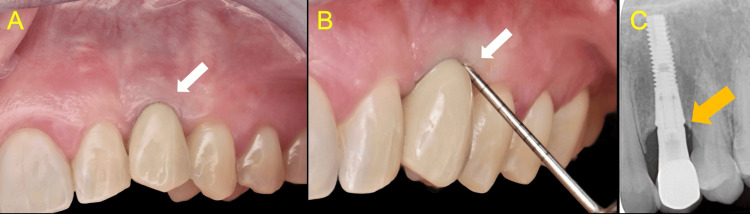
Three-year follow-up A, B: Clinical views showing healthy peri-implant soft tissues with no signs of inflammation, absence of bleeding on probing, and probing depths of 1–2 mm (white arrows) C: Periapical radiograph revealing stable bone levels (orange arrow)

The screw access hole was sealed using a SilverPlug cone and a top layer of micro-hybrid composite resin. Occlusal adjustments were performed as necessary, comprehensive oral hygiene instructions were provided, and the patient was discharged. In the six-month, one-year, and three-year re-evaluation appointments, the site appeared free of inflammation with no bleeding on probing present, and the peri-implant probing revealed values within the range of 1-3 mm (Figure 15).

## Discussion

This case report highlights both biological and prosthetic complications associated with an implant placed at a young age, which can arise due to continuous jaw growth. Such cases are becoming more frequent, as the dynamics of jaw growth have only been better understood in recent years. Thus, the establishment of comprehensive protocols for revisiting these cases is of great importance [2,3]. Key factors in determining whether an implant should be removed or retained and treated include implant stability and three-dimensional positioning, peri-implant bone conditions, visible gingival symmetry and patient concerns. In this case, although the apico-coronal location of the implant platform was deemed excessively deep, its bucco-lingual location was favorable. Additionally, the presented bone stability around the implant, along with the acceptable gingival symmetry, led us to the decision to avoid implant removal and instead focus on addressing the patient's main concern, which was the resolution of the persistent inflammation of the peri-implant soft tissues.

The observed bone stability can be attributed to the original dimensions of the core abutment supporting the prosthesis. Its tall design protected the peri-implant bone by maintaining the cell inflammatory infiltrate at a safe distance from the bone crest. In agreement with numerous studies, it is crucial to protect the deep zone by utilizing tall shoulders and placing the micro-gap, present in the abutment’s shoulder margin, away from the bone crest [4].

The absence of bone loss around the implant dictated biofilm removal confined to the supra-structure and supra-crestal soft tissue. The latter was treated with soft tissue resurfacing to remove the pocket epithelium, followed by application of oxygen- and lactoferrin-releasing blue®m oral gel to control the bacteria and promote the wound healing process. A fundamental step in resolving peri-implant inflammation involves not only proper disinfection of the tissue but also appropriate management of the prosthetic components [5].

The retrieved abutment was found to be contaminated, with open margins and excess cement. The latter is a well-documented cause of peri-implant inflammation, which can occur even years after prosthesis installation, a phenomenon known as “cement sepsis” [6]. The prosthesis exhibited cracks and was made from porcelain material, a material known to be non-biocompatible for the epithelium. When extending sub-gingivally, porcelain impedes the formation of the epithelial attachment, which is crucial as it provides for the superior layer of the mucosal barrier that protects the peri-implant bone from the biofilm present in the peri-implant sulcus. A strong epithelial attachment/soft tissue barrier plays an essential role in preventing bacterial down-growth and ensuring both aesthetic and structural stability of the peri-implant soft tissue around the restoration [7,8]. 

To address these issues, a new prosthesis was designed. A new concave titanium abutment with a 4 mm tall shoulder and a narrow emergence angle was selected to favor soft tissue response in the deep zone [9]. A monolithic zirconia crown was fabricated and cemented extra-orally onto the abutment. The highly polished, non-glazed zirconia in the sub-gingival area facilitates epithelial attachment on its surface and reduces plaque accumulation [10]. The screw-cemented design of the prosthesis allows effective control of cement excess, thereby minimizing the risk of cement sepsis [11].

The cervical zone of the prosthesis comprised a slightly convex emergence shape with a steep emergence angle, following the principles of cervical zone design [9,12]. The prosthesis cervical zone matched in shape the cervical zone of the soft tissue, as this was formed by the anatomical healer used for this purpose. This is also a key factor, as it facilitates easy and efficient circumferential plaque control by the patient. The emergence and cervical profiles in this case were conditioned using an anatomical healer [13]. While other methods, such as the dynamic compression technique, could have been used [14], we chose this methodology due to its simplicity, cost-effectiveness, and the advantage of not requiring numerous abutment connections/disconnections. The latter is known to lead to both hard and soft tissue recession [15].

It is crucial to highlight that both the soft tissue and the prosthesis in this case were treated with oxygen- and lactoferrin-releasing agents, rather than chlorhexidine, which remains widely used in clinical practice. The use of chlorhexidine is associated with numerous well-documented adverse effects, including pigmentation of oral soft tissues and teeth, hypersensitivity reactions, taste disturbances, burning sensation, mucosal ulcerations or erosions, and paraesthesia [16]. Furthermore, chlorhexidine exhibits severe cytotoxic properties. Even when applied in low dosages, it can significantly reduce the viability and migration of human fibroblasts, osteoblasts, gingival keratinocytes, and PRF membranes, thus impairing wound healing [16,17]. It is of great importance that a seven-day use of chlorhexidine mouthwash has been shown to alter the oral microbiome, promote a shift to an acidic environment, and reduce the amount of the oral nitrate-reducing bacteria, which play a crucial role in cardiovascular health [18]. Therefore, the application of chlorhexidine on healing abutments, provisional, or final restorations should be reconsidered. Experimental evidence suggests that it may hinder the important junctional epithelium attachment on the surfaces of the supra-structures used both at the temporarization and final stages, as chlorhexidine has been found to inhibit epithelial cell attachment and proliferation on the surface of implant provisional materials [8].

In contrast, oxygen- and lactoferrin-releasing agents have been shown to promote both antibacterial and wound healing benefits. By delivering in a controlled and safe manner a small amount of oxygen directly to the affected area, topical oxygen therapy enhances soft tissue healing, inhibits the growth of anaerobic bacteria, creates an unfavorable environment for pathogens, and supports immune cell function. This not only decreases the microbial load but also stimulates key biological processes essential for tissue repair, including neovascularization, collagen synthesis, and epithelial regeneration. Lactoferrin complements these effects through supporting angiogenesis, fibroblast proliferation, collagen production and keratinocyte migration [16]. Experimental studies have demonstrated that the topical use of these agents accelerates the healing of standardized surgical skin wounds in rats, with increased angiogenesis and better collagen fiber formation [19], while treatment of human gingival fibroblasts in vitro significantly promotes cell viability and proliferation, preserves cytoskeletal integrity and provides anti-inflammatory and antioxidative effects [20].

Together, topical application of oxygen and lactoferrin promotes microbial control, soft tissue healing and bone regeneration, contributing to a balanced symbiotic peri-implant environment that supports the long-term implant stability and success in clinical practice [16].

Although the outcomes are promising, several limitations of this case report should be acknowledged. The study involves a single case, so the findings cannot be generalized to a larger population without careful consideration. Furthermore, the absence of a control group prevents a comparative evaluation of the treatment. The follow-up period may also be insufficient to fully assess the long-term stability and success of the therapy. 

## Conclusions

The presented case highlights the importance of diagnosing, understanding and managing complications that may arise after implant rehabilitation in growing patients. Infraposition and prosthetic design flaws, such as poor abutment-crown margins, residual cement and presence of non-biocompatible materials subgingivally, can compromise peri-implant tissue health, even in the absence of bone loss.

A biologically driven approach, based on preservation of supracrestal topography, soft tissue re-adherence, and the topical use of oxygen and lactoferrin, resulted in the effective resolution of chronic inflammation and the creation of a stable peri-implant soft tissue seal. Within the limitations of a case report, it can be concluded that proper supra-structure designs, combined with innovative bioactive agents for bacteria control and tissue healing enhancement, can help clinicians re-establish a symbiotic environment and achieve successful results in compromised, complex implant cases. Future studies involving larger sample sizes, control groups, and longer follow-up durations are required to confirm the effectiveness and reproducibility of the treatment strategies described.
